# Intussusception Related to Small Intestinal Lipomas: A Case Report and Review of the Literature

**DOI:** 10.3389/fsurg.2022.915114

**Published:** 2022-06-30

**Authors:** Qiang Hu, Jinfeng Wu, Yuanshui Sun

**Affiliations:** Department of General Surgery, Tongde Hospital of Zhejiang Province, Hangzhou, China

**Keywords:** small intestinal lipoma, intussusception, abdominal pain, surgery, case report

## Abstract

**Introduction:**

Adult intussusception is a rare disease that is difficult to diagnose and treat and is even rarer when it is caused by a lipoma of the small intestine. We reported a case of a small intestine lipoma combined with intussusception, which can guide people in future clinical work.

**Case Presentation:**

A 51-year-old female was admitted to the hospital with “abdominal pain for 1 month.” Enhanced computed tomography (CT) of the abdomen suggested a lipoma in the left lower quadrant and a proximal intussusception. After excluding surgical contraindications, laparoscopic exploration was performed on the second day of admission, which showed a small amount of ascites in the abdominal cavity, a small intestine–small intestine-type intussusception about 20 cm from the ileocecal area and about 140 cm from the ileocecal area, and a mass of about 2×4 cm that was palpable by laparoscopic intestinal forceps, which was protruded into the intestinal cavity with a soft texture and sound mobility. A 5 cm-long incision was made above the mass to dissect into the abdomen layer by layer, and the diseased intestine was dislodged outside the abdominal cavity with oval forceps. The intestine was reduced by hand and observed for half an hour after reduction, and the blood circulation and peristalsis of the intestine were observed to be still sound. The intestine was dissected at 2 cm from the upper and lower margins of the mass using linear anastomosis to operate small intestine side-to-side anastomosis. The intestine was opened concurrently and closed with a linear anastomosis, using 3-0 absorbable thread to reinforce anastomosis intermittently. The procedure went smoothly, and the patient was discharged on the 5th postoperative day.

**Conclusion:**

A small intestinal lipoma combined with small intestinal intussusception is rare in clinical practice and needs to be diagnosed by asking history detailedly, physical examination, and relevant ancillary tests such as abdominal CT. Laparoscopic-assisted small incision surgery for adult intussusception combines the advantages of laparoscopic surgery and laparotomy, operating simply and easily.

## Introduction

Adult intussusception is a rare disease that is more difficult to diagnose and treat, which is mostly caused by organic lesions of the small intestine, such as small bowel tumors, diverticula, and polyps (1). Small intestinal intussusception caused by a small intestinal lipoma is rare clinically, and the lipoma is usually detected after it has caused intussusception. Therefore, the diagnosis of intussusception is quite challenging (2).

## Case Presentation

**Figure 1:**
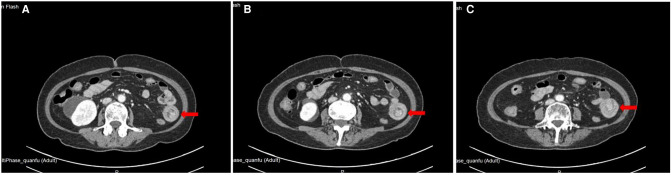
Location of small intestinal intussusception in abdominal enhanced CT.

**Figure 2:**
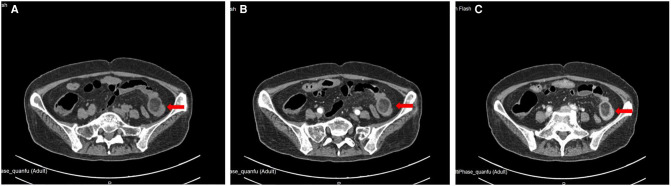
Location of small intestinal tumor in abdominal enhanced CT. (**A**) plain scan; (**B**) arterial phase; (**C**) venous phase.

A 51-year-old female was admitted to the hospital with “abdominal pain for 1 month.” According to the patient, she had abdominal pain after eating before January; the pain was paroxysmal dull pain with short duration and relieved spontaneously after about 5 min; she had no fever and chills, no nausea and vomiting, no obvious radiating pain, and no cessation of anal venting and defecation; the patient was not treated at that time so the symptoms were not relieved; hence, she came to our hospital for treatment. The following was observed upon physical examination: T 37.2°C, P 95 times/min, R 19 times/min, BP 126/95  mmHg, abdominal bulge, left lower abdominal tenderness, no significant rebound pain, no muscle tension, intestinal sounds 3–4 times/min, and negative shifting dullness. The following was observed upon laboratory examination: white blood cell 8.6×10^9^/L, neutrophils 88.2%, hemoglobin 76 g/L, ultrasensitive C-reactive protein 2.8 mg/L, procalcitonin (PCT) 0.08 ng/ml, albumin (ALB) 32.5 g/L. Imaging examination shows the following: enhanced CT of the abdomen suggested a lipoma in the left lower abdomen in the small intestine with proximal intussusception (Figures 1, 2). The patient’s past history indicated that she was in good health.

**Figure 3:**
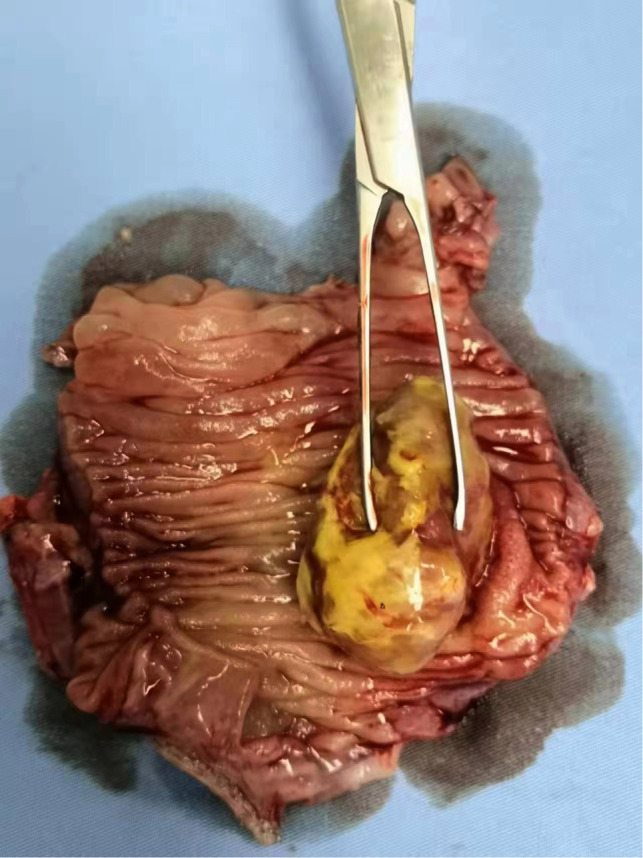
Image showing the cut open specimen and a yellow swelling of about 2×4 cm.

**Figure 4:**
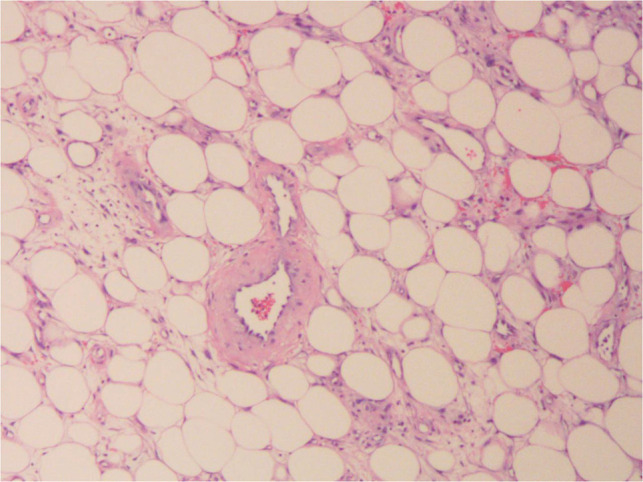
Postoperative pathology suggesting localized adipose tissue neoplastic hyperplasia in the intestinal wall, consistent with a lipoma (HE×100).

Because the patient has no obvious contraindications to surgery or anesthesia, laparoscopic exploration was performed on the second day of admission, which showed a small amount of ascites in the abdominal cavity, a small intestine–small intestine-type intussusception about 20 cm from the ileocecal area. An incision of about 5 cm in length was made above the mass to dissect it into the abdomen layer by layer, and the diseased intestine was dislodged from the abdominal cavity with oval forceps. After observing, the color of the intussusception was slightly red and no obvious necrosis or rupture was seen. Then, the intestine was reduced by hand and observed for half an hour after reduction, and the blood circulation and peristalsis of the intestine were found to be still sound. The intestine was opened concurrently at 2 cm from the upper and lower margins of the mass and closed with a linear anastomosis, and the common opening was closed with a linear anastomosis using 3-0 absorbable thread to reinforce anastomosis intermittently. The final examination of anastomosis showed its patency and sound blood supply. The specimen was cut open, and a yellow swelling of about 2×4 cm was seen (Figure 3). The patient developed anal discharge and was given a liquid diet on the second postoperative day; on the third postoperative day, the patient developed anal bowel movements and was given a semiliquid diet; and on the fifth postoperative day, the patient was discharged from the hospital after resuming a normal diet. Postoperative pathology suggested localized adipose tissue neoplastic hyperplasia in the intestinal wall, consistent with a lipoma (Figure 4). Immunohistochemistry and oncogene examination shows the following: S-100(+), SOX10(−), β-catenin(+), P53(+), Ki-67(+,5%), CD34(+), ERG(+), SMA(+), Desmin(+), Vim(+), PCK(−), CD68(+), and CD163(+). The recovery was good, with no complications at the 2-week postoperative follow-up by telephone.

## Discussion

Small intestinal intussusception refers to a section of the small intestine that is overlapped into the adjacent small intestine, and adult small intestinal intussusception is clinically rare with a low incidence of 5%–10%, accounting for 1%–5% of adult intestinal obstruction (3). It is different from children's intussusception that 70%–90% of small intestinal intussusception in adults is due to the pathological causes, mainly secondary to small bowel occupying, which include lipomas, gastrointestinal mesenchymal tumors, polyps, small bowel metastases, and malignant melanoma, and nonoccupying lesions, such as Meckel's diverticulum and inflammatory lesions of the small bowel (4). In addition, only 8%–20% of adult intussusceptions are idiopathic (5). The early clinical manifestations of intussusception in adults include abdominal distension, nausea, abdominal pain, and bloody stools, and the late manifestations are signs of peritoneal irritation and intestinal obstruction; however, these manifestations lack specificity (6). Small intestinal tumors account for only 1%–2% of all GI tumors, of which approximately 30% are benign lesions (7). Small intestinal lipomas account for 2.6% of all benign GI tumors (8). Lipomas tend to occur in the subcutaneous tissue and less frequently in the GI tract. Gastrointestinal lipomas are usually asymptomatic and can sometimes be associated with abdominal pain, blood in the stool, and obstruction symptoms. Similar to intussusception, a small intestinal lipoma is difficult to diagnose by clinical symptoms only.

The mechanism by which intussusception occurs is unclear, but it is generally accepted that any lesion or stimulus that alters normal bowel motility may initiate the intussusception process (9). Intussusception occurs when the proximal intestine sets into the distal intestine adjacent to it, and the trigger point for intussusception is mostly at the junction of the lesion or the more mobile intestine and the more fixed intestine (10).

The diagnosis of a small intestinal lipoma mainly depends on imaging, and barium gastrointestinal imaging has some value, which shows round, ovoid, or lobulated filling defects in the intestinal lumen of the small intestine with a smooth border, no tip, variable morphology under pressure and normal local intestinal peristalsis and mucosa (11). However, the gastrointestinal barium meal imaging examination shows more overlap of the intestinal cavity, which is easy to miss the diagnosis when the intestinal canal of the lesion is poorly exposed; it is difficult to diagnose a lipoma when combined with intestinal obstruction and intestinal overlap, and it is difficult to distinguish it from other filling defects in the intestinal cavity, which is easy to miss the diagnosis when the lipoma is small. Thus, this examination method has its limitations (12). Ultrasonography has little diagnostic value for small bowel lesions because of the presence of gas in the lumen of the small intestine, which interferes with ultrasonography (13). The small intestinal lipoma has a specific lipid component, and CT examination of the abdomen showed a rounded low-density shadow in the intestinal lumen with CT values of −70 to 120 HU and no enhancement after enhanced abdominal CT (14). Fang et al. reported that a 3D CT examination of the small intestine could establish a reliable preoperative diagnosis by obtaining characteristic images of lipomas (15), even though small intestinal tumors, including lipomas, are difficult to detect early unless intussusception or intestinal obstruction occurs. Abdominal CT is essential in the diagnosis of intussusception, which can present as a “target sign” or a concentric circle-like presentation (16). A double intestinal canal sign can be seen when the long axis of intussusception is parallel to the scanned level, which has a high value in the preoperative examination of intussusception in adults (17). Therefore, the diagnosis of small intestinal lipomas combined with small intestinal intussusception is made by asking for history detailedly, combined with physical examination and relevant ancillary tests such as abdominal CT.

Small intestinal intussusception in adults is mainly caused by organic lesions of the small intestine, and once the diagnosis is clear, surgical treatment is mainly used to remove the lesion or the entrapped segment of the intestine, and rapid frozen section examination is required to exclude malignant tumors of the small intestine if the tumor cannot be characterized intraoperatively (18, 19). To date, a unanimous agreement on which is the best treatment modality does not exist. The traditional surgical method is open surgery with the advantages of simple operation and mature technology; however, in recent years, laparoscopic surgery has developed rapidly with the advantages of safety, reliability, minimally invasive, wide surgical field, rapid postoperative recovery, and few postoperative complications, which are in line with the enhanced recovery after surgery, which is being carried out more and more widely in gastrointestinal surgical diseases (20, 21). In small intestinal surgery, the lack of support points during laparoscopic surgery is not easy to operate on due to the vibrate intestinal mobility, and the long operation time under total laparoscopy is not conducive to the patient's postoperative recovery. Some studies have considered the presence of limited working space for the tension of the small bowel, and they preferred a laparotomic rather than a laparoscopic approach (22). We combined the advantages of laparoscopic surgery and laparotomy using laparoscopic surgery when exploring the lesion and locating the incision. After the lesion was identified laparoscopically, a small 5-cm auxiliary incision was made at the nearest part of the lesion; because of the large mobility of the small intestine, the diseased intestine could be pulled out of the abdominal cavity by oval forceps, and intestinal resection and anastomosis surgery was performed under direct extraabdominal vision, which was a convenient operation.

## Conclusion

A small intestinal lipoma combined with small intestinal intussusception is rare in clinical practice and needs to be diagnosed by asking for the history detailedly, physical examination, and relevant ancillary tests such as abdominal CT. Laparoscopic-assisted small incision surgery for adult intussusception combines the advantages of laparoscopic surgery and laparotomy, operating simply and easily.

## Data Availability

The original contributions presented in the study are included in the article/Supplementary Material, further inquiries can be directed to the corresponding author.
